# Management of Iron Overload in Resource Poor Nations: A Systematic Review of Phlebotomy and Natural Chelators

**DOI:** 10.1155/2020/4084538

**Published:** 2020-01-27

**Authors:** Orish Ebere Orisakwe, Cecilia Nwadiuto Amadi, Chiara Frazzoli

**Affiliations:** ^1^Department of Experimental Pharmacology & Toxicology, Faculty of Pharmacy, University of Port Harcourt Rivers State, Nigeria; ^2^World Bank Africa Centre of Excellence in Public Health and Toxicological Research (PUTOR), University of Port Harcourt, PMB, 5323 Port Harcourt, Rivers State, Nigeria; ^3^Department for Cardiovascular, Dysmetabolic and Aging Diseases, Istituto Superiore di Sanità, Rome, Italy

## Abstract

Iron is an essential element and the most abundant trace metal in the body involved in oxygen transport and oxygen sensing, electron transfer, energy metabolism, and DNA synthesis. Excess labile and unchelated iron can catalyze the formation of tissue-damaging radicals and induce oxidative stress. English abstracts were identified in PubMed and Google Scholar using multiple and various search terms based on defined inclusion and exclusion criteria. Full-length articles were selected for systematic review, and secondary and tertiary references were developed. Although bloodletting or phlebotomy remains the gold standard in the management of iron overload, this systematic review is an updated account of the pitfalls of phlebotomy and classical synthetic chelators with scientific justification for the use of natural iron chelators of dietary origin in resource-poor nations.

## 1. Introduction

Iron is an essential nutrient and a vital moiety of many proteins like iron-sulfur clusters (found in complex I and II, which are important for redox reactions involved in respiration, cellular energy metabolism, DNA synthesis, cell growth, and proliferation), heme moieties of cytochromes *b*, *c, *and cytochrome P450 that partake in oxidative phosphorylation and xenobiotics detoxification [1–4]. Iron is also utilized in other redox reactions within the cells [5, 6]. Incidentally, the inherent capacity of iron to revolve from its Fe^2+^–Fe^3+^ which enables its loss or gain of electrons, considered vital in electron transport is the same feature that is implicated in its cellular toxicity via the generation of cytotoxic reactive oxygen species ROS by donating electrons to oxygen.

In man, an estimated four grams of iron is found in a heme form within hemoproteins (80% of iron), nonheme form distributed between storage (ferritin and hemosiderin) and transport proteins (transferrin). Iron absorption is predominantly in the duodenum where dietary nonheme trivalent iron is reduced to divalent iron by the ferrireductase duodenal cytochrome b. The Divalent Metal Transporter 1 DMT1, sequesters the divalent iron in the apical membrane of enterocytes to become a part of the Labile Iron Pool. In man, there are no regulatory mechanisms for iron excretion, however, the body burden of iron is largely controlled by regulatory mechanisms for absorption from the gut [7]. There should, therefore, be a balance between iron uptake, usage, and storage to maintain a tightly regulated intracellular iron concentration at all times [8]. Iron is indispensable for life but too much of anything is bad. Unchelated or nontransferrin bound iron is known to catalyze the formation of free radicals such as hydroxyl and other radical species. Iron overload as seen in hemochromatosis is an abnormal uptake of iron resulting in its accumulation in various organ systems with the attendant exaggerated formation of free radicals and consequent damage [9]. Derangement in iron homeostasis has been implicated in various diseases from neurological disorders such as Alzheimer's and Parkinson's diseases to metabolic syndrome. As a redox active metal, iron is involved in the oxidation-reduction reactions that generate free radicals [10], linked with the catalytic decomposition of hydrogen peroxide (Fenton reaction) leading to the formation of reactive hydroxyl radicals causing damage to biomolecules, including lipids, proteins, and DNA [11].

For many years, phlebotomy has been employed as the gold standard for the management of iron overload in hereditary hemochromatosis. However, phlebotomy is not usually optimal in some conditions of iron overload, especially anemia. Even the suggested alternative like erythrocytapheresis also called automated red blood cell exchange (aRBCX) may not be feasible in resource-poor countries of Sub-Sahara Africa (SSA) due to the lack of infrastructure. Use of iron chelators is an alternative where phlebotomy is not feasible [12]. The evaluation of the effectiveness, safety, and cost of chelation treatment in the management of transfusion siderosis in sickle-cell disease with synthetic or classical chelators reported an absence of evidence regarding their effectiveness [13]. In view of the prohibitive cost, nonavailability and wide range of adverse effects of classical iron chelators may contribute to resorting to natural chelators in the management of iron overload in resource-poor nations. This systematic review seeks to provide evidence for the multimodal mechanistic considerations and beneficial roles of natural iron chelators in the management of various conditions of iron overload in resource-poor nations. This is an updated account of natural iron chelators including new experimental agents expected to be applicable as a deferration agents in various iron overload diseases. As much as possible this paper has also highlighted the relevance and preference of natural iron chelators over phlebotomy.

## 2. Methodology

Multiple online interactive searches in the databases of PUBMED, GOOGLE SCHOLAR, and SCOPUS for original research using terms such as “natural iron chelators”, “management of iron overload”, “phlebotomy and iron chelators”, “treatment of iron poisoning”, “natural antidotes for iron poisoning”, “iron chelation in metabolic syndrome”, “use of plant extracts in the treatment of iron toxicity”, “foods and supplements for treating iron toxicity”, etc. Search results were screened, full texts obtained, inclusion and exclusion criteria applied to determine the suitability of articles used in this review. Studies that reported beneficial dietary effects of whole, parts or extracts of herbal plants on iron overload were included, and studies were excluded if the material used is a nonsupplemental synthetic drug or chemical agent (except for purposes of comparison). Articles in any other language other than English were excluded.

## 3. Results and Discussion

### 3.1. Search Results

**Figure 1:**
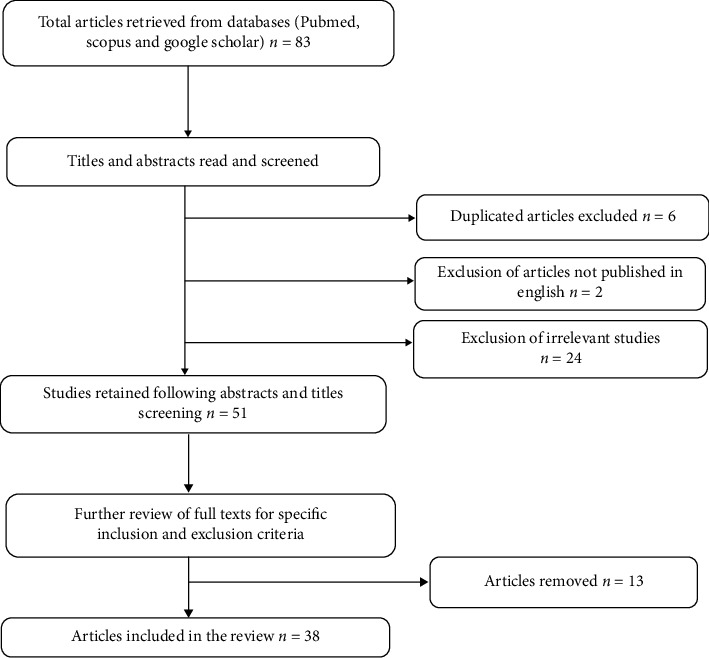
Study selection flow diagram.

A total of 83 studies were found in the initial search. After screening their titles and abstracts, 32 articles were excluded leaving 51 articles for further review. The articles were excluded based on being relevant or not, twenty-four articles were not relevant (*n* = 24), not available in English (*n* = 2) and duplications (*n* = 6). Further review of the full texts of the remaining articles with the application of the inclusion and exclusion criteria resulted in the exclusion of 13 additional articles, leaving 38 studies that were included in this review (Figure 1).

### 3.2. Some Diseases Associated with Iron Overload and Their Prevalence in Sub-Sahara Africa (SSA)

#### 3.2.1. Neurodegenerative Diseases

Iron overload in the brain is now implicated in a myriad of neurodegenerative diseases like Alzheimer's disease, Parkinson's disease, Huntington disease, Friedreich ataxia, and amyotrophic lateral sclerosis. The iron accumulation in certain brain regions like the substantia nigra in Parkinson's disease trigger the generation of reactive oxygen species and intracellular *α*-synuclein aggregation, culminating in the oxidative neuronal destruction of this brain area [14]. Brain iron dyshomeostasis is also associated with the activation of the N-methyl-D-aspartic acid receptor, a signaling neurotoxicity cascade involving the enzyme nitric oxide synthase and adaptor proteins that interact with ferroportin, such as the divalent metal transporter-1 [15]. In the absence of high-quality prospective cohort studies, which employ internationally-validated criteria to help map the epidemiology of neurodegenerative diseases in SSA coupled with poor record keeping, it could be said that there is a paucity of information on the incidence of neurodegenerative diseases in SSA. Parkinsonian disorders like any other neurodegenerative disease are under-diagnosed in Nigeria with a crude estimate lower (10–249/100 000) than European data (65.6–12 500/100 000). Heavy metals through occupational exposure like blacksmithing and potable water have been implicated in the cases recorded in Nigeria [16]. The estimated crude prevalence of Parkinsonian disorders in Nigeria was lower (10–249/100 000) compared to studies published in Europe (65.6–12 500/100 000) [17].

#### 3.2.2. Cancer

At least 100 000 new cases of cancer occur annually in Nigeria, with high case fatality ratio [18]. Nigeria contributed about 15% of the estimated 681 000 new cases of cancer that occurred in Africa in 2008 [19]. The few or no case reports of cancer survivors in Nigeria delineate poor management. The depleted levels of the protein ferroportin (only known protein to eliminate iron from the cells) has been linked with the incidence of most aggressive and recurring cancers such as breast cancer [20]. A report of cancer incidence from two regions in Nigeria revealed that the most common cancers in women were cancer of the breast and cervix; and cancer of the prostate in Nigerian men [21]. Of note the increasing incidence of breast cancer is an aggressive cancer dependent on iron in recent times [21].

#### 3.2.3. Metabolic Syndrome

Some countries in SSA are currently undergoing a rapid epidemiological transition to an increasing number of metabolic disorders [22], with suggestions like demographic changes such as aging, and the undesirable risk factors such as obesity and sedentary lifestyles as the causative factors [23–26].

According to the National Cholesterol Education Program Third Adult Treatment Panel (NCEP ATP-III) ATPIII, the International Diabetes Federation IDF, and the World Health Organization definition WHO definitions, the prevalence of cardiometabolic syndrome (a complex cluster of risk factors for cardiovascular disease, diabetes, dyslipidaemia, hypertension, and obesity) in Nigeria as follows 27.9% (NCEP ATP-III), 28.1% (IDF), and 31.7% (WHO). These values are considered higher than the prevalence of 19.1% using ATPII criteria in Canada [27] and comparable to the prevalence of 33.5% in Australia according to the IDF definition [28], and unadjusted prevalence of 34.1% in the USA based on the ATPIII criteria. Amidst inadequate prevalence studies on cardiometabolic syndrome in Sub-Saharan Africa, available evidence suggests that Nigeria has the highest frequency of cardiometabolic syndrome among the Sub-Saharans probably due to the growing economic strength and the degree of western influence [22, 29].

#### 3.2.4. Sickle Cell Disease

Sickle-cell disease is a qualitative haemoglobinopathy associated with mutations in the HBB gene, resulting from point mutations that change the sixth amino acid in the beta-haemoglobin chain from glutamic acid to valine (Glu6Val). It is characterized by chronic haemolytic anaemia, intermittent vaso-occlusive events, tissue Ischaemia (leading to acute and chronic pain), ischaemic and haemorrhagic stroke, acute chest syndrome, splenic sequestration, aplastic crises, bacterial sepsis resulting from hyposplenia with chronic morbidities such as cerebrovascular disease, pulmonary hypertension, osteonecrosis, nephropathy, and organ failure. Despite its predominant prevalence in Africa, the sickle-cell disease remains an orphan disease with the lack of specific funds for its management and research [30]. Although when performed according to stipulated guidelines appropriate blood transfusions may prevent and treat sickle cell disease associated complications [31], blood transfusion requirements in sickle-cell disease inevitably lead to increased body iron burden and, consequently, iron-related organ damage and complications, notably hepatic damage and mortality [32]. Sickle-cell disease is associated with a chronic inflammatory state in children with the hallmark of high sensitivity C-reactive protein, a marker of inflammation and vaso-occlusive-crises leading to hospitalization [33, 34].

There is a high prevalence of sickle cell trait (10–45%) [35–37] in SSA and 2.39% sickle cell disease in Nigeria [38] due to the survival advantage conferred by the sickle cell trait against *Plasmodium falciparum*.

## 4. Management of Iron Overload

### 4.1. Phlebotomy: Gold Standard and Pitfalls

Phlebotomy or bloodletting since its earliest use in the 1950s seems to have been the gold standard in the management of hemochromatosis or iron overload with treatment often commenced when serum ferritin levels exceed the normal range [39]. Phlebotomy is the induction of a mildly iron-deficient state. The determination of the severity of iron overload and monitoring the response to treatment usually require a battery of tests *viz* laboratory quantification of serum ferritin concentrations, magnetic resonance imaging (MRI) to assess the liver and cardiac iron levels, and, in some cases, liver biopsy [7, 40]. It is usually advisable to avoid iron deficiency with lower serum ferritin levels since this may be associated with unnecessary and worrisome symptoms or, paradoxically, lead to further hepcidin (type II acute-phase protein that mediates the hypoferremia associated with infection and inflammation) depression and increased iron absorption during therapeutic phlebotomy [39]. Generally, phlebotomy is a delicate titration requiring in-depth knowledge of the patient's serum ferritin levels by the physician that cannot be handled by a lay-person or outside the hospital setting.

Therapeutic phlebotomy is contraindicated in conditions like severe anemia, cardiac failure, or poor tolerance and in all these iron chelators have been considered as an alternative. Given that for ethical reasons the efficacy of phlebotomy is yet to be validated in controlled studies and its survival benefits remain hitherto not evaluated in patients with hemochromatosis [39]. Phlebotomy tends to improve transaminase levels, skin pigmentation, and hepatic fibrosis but seem to have no beneficial effects on life expectancy in hemochromatosis-related hypogonadism, cirrhosis, destructive arthritis, and insulin-dependent diabetes [41]. Since dietary absorption of divalent metals, including iron, require the same transporter (DMT1), homeostasis of the other metals will be continuously abnormal in patients who undergo phlebotomy [42, 43].

### 4.2. Dietary Sources of Iron-Chelators

The participation of unbound or loosely chelated iron in intractable generation of ROS and tissue damage remains a common feature in iron overload related diseases. Therefore, effective scavenging of excess iron is a plausible means to restrain and quell free radical-mediated tissue damage. Iron chelation is gaining traction in the management of various iron-related diseases. Chelators will not only remove iron from the body but also scavenge and firmly bind free iron to prevent the generation of ROS [44, 45]. Classical chelation is widely used in the treatment of iron loading anaemias but because of its cost, inconvenience, monitoring requirements, and untoward effects, newer chelating agents especially of dietary sources that are cheaper and more readily available might provide effective alternatives for this clinically consequential and common group of disorders.

An ideal chelator of redox active metals should tightly bind Fe^3+^, have low molecular weight, possess lipophilicity to penetrate the blood–brain barrier in the case of management of neurodegenerative diseases and possess minimal toxicity. Interestingly, one of the most notable iron chelators desferrioxamine is of natural origin. Desferrioxamine is produced by the *Streptomyces *species [46], with a molecular structure that consists of multiple hydroxyl and carbonyl groups that can chelate iron in a 1 : 1 ratio. Its relevance in iron overload management lies in its specific preference in the binding of iron over calcium to protect myocytes against peroxide-induced damage [47].

Their chemical diversity notwithstanding, typically iron chelators contain oxygen, nitrogen or sulfur-donor atoms that form coordinate bonds with bound iron. These donor atoms namely oxygen, nitrogen or sulfur of the ligand affect the preference of the chelator for either the Fe^2+^ or Fe^3+^ oxidation states [48]. Usually, Fe^2+^ chelators possess nitrogen and sulfur donor atoms (so-called ‘soft' donor atoms) which also have a high affinity for other biologically important divalent metals such as Cu^2+^ and Zn^2+^ [49]. An ideal iron chelator must also effectively compete with the biological ligands that normally bind iron; therefore, the affinity of chelators for iron, and their stoichiometry of iron binding will greatly impact their activity as therapeutic agents [50, 51]. Iron chelation is undoubtedly of immense benefit in the management of iron overload in man but can play other useful roles in diseases mediated by oxidative stress such as ischemia-reperfusion injury [52], liver infectious [53], and neurologic diseases [54], diabetes, inflammation [55–57], and atherosclerosis [58]. There is a need to extensively explore natural compounds derived from microorganisms siderophores and plants.

Literature is replete with studies that lend credence to the fact that foods containing plant polyphenols and flavonoids may have benefits not only as potent antioxidants but also as iron chelators [59–62] (Tables 1 and 2). These flavonoid-rich foods according to their subclasses include flavanols (examples of rich sources: teas and red wine), flavanone (citrus foods), flavones (fruit skins, peppers, and leafy vegetables), isoflavones (soy foods), flavonols (leeks, onions, leafy vegetables, and tomatoes), anthocyanidins (berries) and proanthocyanidins (apples, chocolate, and nuts) [63]. A common chemical feature of proanthocyanidins, epicatechins, flavonol, and anthocyanin is the iron-binding motif like the catechol moiety that is a known iron-binding element of microbial siderophores [64].

#### 4.2.1. Spices/Turmeric

Turmeric is a spice from the root of a turmeric plant (*Curcuma longa*) with yellow colored active ingredient called curcumin. Iron chelation is an inherent property of curcumin that has been employed in the management of cancer [44, 65–67]. Both *in vitro *studies involving liver cells treated with curcumin (one of the naturally occurring iron chelators) and *in vivo *studies using mouse model of thalassemia, exhibited fingerprints of iron depletion, which included decreases in the iron-storage protein ferritin, increases in transferrin receptor 1, repressed synthesis of hepcidin and activation of iron regulatory proteins [68]. Curcumin crosses the blood-brain barrier to exert its antioxidant and iron-chelating properties in the brain [69–71]. Curcumin also has shown neuroprotection capability in a Parkinson disease model [72] reducing both oxidative damage and amyloid pathology in an Alzheimer disease model [73].

#### 4.2.2. Staple Crops

Polyphenols abundant in different types of foods like wheat, potato, soybean, sorghum, and common beans are known inhibitors of iron bioavailability [74]. Several workers have demonstrated strong binding capacity of polyphenols with iron [75–77]. *In vitro* and *in vivo *studies with whole colored beans and the seed coats of colored beans have regularly shown polyphenol mediated impairment of iron bioavailability [78, 79] and this inhibition has been demonstrated to be due to the presence of polyphenols in the seed coat. Similarly, the antioxidant activities polyphenol-rich extracts obtained from both green tea and grape seed is known to accentuated by their iron chelation potency [80, 81] Polyphenols impede nonheme iron absorption by decreasing basolateral iron exit rather than by reducing apical iron import in intestinal cells [82].

Phytate in soy protein is a strong inhibitor of nonheme iron absorption in humans [83, 84] Postmenopausal women who are at risk of excess iron may benefit from dietary soy protein to reduce iron stores, and lower cardiovascular risk [85]. Reduced iron stores as evinced by serum ferritin concentrations, reduced serum iron, and transferrin saturation were observed in postmenopausal women after six-week consumption of commercially prepared powders of soy protein with native phytic acid [85].

#### 4.2.3. Teas

Phyto-polyphenols, like epigallocatechin gallate (EGCG), are also natural iron chelators. Epigallocatechin gallate (EGCG), (one cup of tea contains 30–130 mg EGCG) has several metal binding sites in its structure. The antioxidant/neuroprotective activity of green tea catechins like EGCG is linked to their iron chelation properties [86]. Recent studies have shown that the phenolic hydroxyl groups on the aromatic rings of EGCG confer the antioxidant and iron-chelating activities [44, 87, 88].

EGCG has been demonstrated to be neuroprotective in experimental models of Parkinson disease [89, 90], Alzheimer's disease [91], and amyotrophic lateral sclerosis [92]. Green tea catechins in addition to being scavengers of free radicals have well-defined metal-chelating properties, in their 3′,4′-dihydroxyl group in the B ring as well as the gallate group [82, 93].

Earlier consideration of beneficial effects of green tea catechin polyphenols hinged mainly on free radical scavenging but at the moment these polyphenols are known to be multifaceted acting compounds that direct numerous cellular neuroprotection/neurorescue mechanisms involving iron chelation, scavenging of oxygen, and nitrogen radical species and activation of protein kinase C signaling pathway and prosurvival genes. Since the green tea catechin polyphenols are not toxic and possess the ability to permeate the blood-brain barrier given their lipophilicity they have been touted for removal of iron from specific brain areas where it preferentially accumulates in neurodegenerative diseases [94]. Heavy metals especially iron is implicated in the activation of redox cycling; thus, iron-chelation therapy should be considered as a valuable strategy for the treatment of neurodegenerative diseases [95].

#### 4.2.4. Berries

The fruits of elderberry, *Sambucus nigra *L., a common wild-growing bush in many parts of Africa are a rich source of cyanidin-based anthocyanins which is a potent iron chelator. The iron chelating potency of “Haschberg extract” majorly attributed to cyanidin-3-glucoside was found to be higher than even known standard iron chelators [96]. Anthocyanins are close derivatives of flavonoids and thus the functional groups responsible for chelation/reduction may be similar.

#### 4.2.5. Citrus/Grape Seed

Gallic acid, catechin, and epigallocatechin gallate (EGCG) are also polyphenols from grape seed extract with potent antioxidant activities attributable to iron chelation [82]. Grape seed extract and epigallocatechin gallate (EGCG) suppress nonheme iron absorption in human intestinal Caco-2 cells [82].

#### 4.2.6. Garcinia kola

*Garcinia Kola* nut, a commonly chewed bitter seed in Nigeria contains kolaviron, a natural bioflavonoid found to be a potent iron chelator in the protection against lipid oxidation in rats [97]. Quercetin binds both Fe^3+^ and Fe^2+^ with even a stronger affinity for Fe^2+^ than ferrozine a well-known Fe^2+^ chelator. At micromolar concentration, even in the presence of the major cellular iron chelators ATP or citrate, quercetin can inhibit iron-promoted Fenton chemistry suggesting that the radical scavenging property of quercetin provides only partial protection against damages mediated by Fenton chemistry. Taken together, the antioxidant activity of quercetin may largely be due to its iron chelation property [98].

In a wide range of cellular and animal models of neurological disorders, catechins have shown their ability to chelate divalent metals in addition to their antioxidant, anti-inflammatory activities in penetrating the blood–brain barrier and eventual protection of neuronal death [99].

#### 4.2.7. Plant Extracts

Silymarin is the flavonoid extract of *Silybum marianum*, or milk thistle with its major active compound as silybin, when consumed with a meal resulted in a considerable reduction in the amount of dietary iron absorbed [100]. In the neutral pH of the duodenum, silybin forms a complex with unchelated ferric iron [101], to hamper its absorption. Silybin is more effective in limiting the postprandial increase in serum iron compared with tea [100]. Vitamin C enhances absorption of nonheme iron [102], and in fact, counteracts the iron-binding effect of tea polyphenols [103]. Silybin consumption has led to the appreciable reduction in the amount of iron absorbed from a single meal, even in the presence of vitamin C [100]. Similarly, another study also reported that the iron-chelating properties of silybin were responsible for the decrease in the body burden of iron in patients with chronic hepatitis C after 12 weeks of oral silybin [104]. *In vivo *iron chelating studies and phenolic profiles of the angel's wings mushroom, *Pleurotus porrigens,* a culinary-medicinal mushroom reported satisfactory potency to chelate excessive iron in mice, potentially offering a new natural alternative to treat patients with iron overload [105].

The hepatoprotective action of methanol extract of *Acacia catechu* heartwood or *Katha* against hepatic damage induced by iron overload in mice is also thought to be by ameliorating the antioxidant defense activities and reductive release of ferritin iron [106]. The ethanolic extract of *Azadirachta indica* tested for free radical scavenging activity by 2,2′-azino-bis-3-ethylbenzothiaziline-6-sulfonic acid (ABTS) and for the reduction of the power of ferric ion Fe^3+^ to ferrous ion Fe^2+^ by ferric reducing antioxidant plasma (FRAP) assay, showed free radical scavenging activities, decreased the redox cycling of ferric ion (Fe^3+^) to ferrous ion Fe^2+^ in dose-dependent manner and a rapid binding of iron [107].

Other phenols (chrysin, puerarin, naringenin, and genistein) and traditional Chinese medicine/herbs (panax ginseng, ginkgo biloba, scutellaria baicalensis Georgi) are also known to have strong iron chelation property [89, 90].

#### 4.2.8. Shrimp

Shrimp shell wastes are rich sources of phenolic compounds [108], with important antioxidative properties [109, 110]. The rationale behind the consumer's preference for natural antioxidants has been accentuated by their multimodal activities and toxicity of synthetic antioxidants [111, 112]. The squid pen powder fermented extract rich in phenolic and amino containing compounds is bioactive rich liquor with beneficial biological functions due to its inherent protein and chitin hydrolysis activity as well as the production of other bioactive materials during fermentation [113]. Serranticin isolated from the squid pen powder is analogous to siderophores (hexacoordinated catecholamine), which are iron chelators [113]. Serranticin may be of pharmacotherapeutic value in the management of diseases related to iron overload [113]. Similarly, parabactin, isolated from *Paracoccus dentrificans* [114], is at least 300% more effective than desferrioxamine, a known chelating agent for iron decorporation, in removing iron from a rodent model [115].

Most polyphenolic compounds notably flavones, isoflavones, stilbenes, flavanones, catechins (flavan-3-ols), chalcones, tannins, and anthocyanidins are known to chelate iron in addition to their antioxidant effects [116]. Flavones such as quercetin [117], rutin [118], gossypetin, myricetin, quercitrin, isoquercitrin [119], and flavonol [120] are the most potent in these regards. In Fanconi anemia and thalassemia, rutin appreciably suppresses free radical production by neutrophils and increased the hemoglobin level [121].

Antioxidant activity [87, 122], regulatory inhibition of mitochondrial monoamine oxidase MAO activity [123, 124], stabilization, and transcriptional activation of iron-dependent HIF-1 [125–127], a selective ability to inhibit protein aggregation and accumulation are some additional beneficial neurotherapeutic properties of iron chelators in iron overload [14, 128]. Iron chelation by catechins affects not only the posttranscriptional regulation of iron homeostasis-related RNAs, but also the induction of genes regulated by the hypoxia induced factor 1 HIF-1, that regulates the physiological responses to low oxygen levels and the pathophysiology of heart attack, cancer, stroke, and chronic lung disease [129]. There is an experimental evidence that dietary iron restriction or iron chelation protects from diabetes and loss of *β*-cell function in the obese mouse [130]. Although low-iron diet significantly ameliorated diabetes in the mice, iron chelation had a more dramatic effect, allowing the obese mice to maintain normal glucose tolerance for at least ten weeks despite no effect on weight [130].

Iron chelation is the laudable alternative in iron overload in transfusional siderosis induced in patients with thalassemia major, and other refractory anaemias [131]. It has also been employed in thalassemia major patients and in other conditions of haemoglobinopathies such as sickle cell anaemia [132, 133]. Sickle cell anemia is most prevalent in Sub-Sahara Africa, especially in equatorial African populations. Children born with such severe haemoglobinopathies like thalassemia major and sickle cell anaemia live on regular transfusions with no consensus on the follow-up, and therapy of the resulting iatrogenic siderosis.

With the contraindication of phlebotomy and the inevitable likelihood of most patients swiftly attaining ferritin levels above 2000 *µ*g/l, iron chelation with desferrioxamine and deferiprone form the mainstays of therapy in developed nations where these iron chelators are available and affordable [134]. The poor oral absorption of desferrioxamine leaves no alternative for its administration but expertise demanding slow intravenous or painful subcutaneous infusions which compromise patients' compliance [135, 136] or not be affordable in resource-poor settings. Ophthalmic and auditory toxicity, bacterial and fungal infections, haematogical changes, allergic and skin reactions, and pulmonary, renal and neurological effects [137] are the daunting side effects of desferrioxamine in addition to the prohibitive cost [138]. Deferiprone the orally active iron chelator is not also devoid of the side effects of desferrioxamine has been shown in patients who reported gastric discomfort, zinc depletion, leukopenia, transient agranulocytosis or transient musculoskeletal, and joint pain. In developed nations, the clinical experience with iron chelation in transfusional siderosis is from thalassaemia patients as shown in literature with very lean mention of sickle cell anaemia which is common in resource poor nations in Sub-Sahara Africa. There is a great need for clinical studies of iron chelation therapy in sickle cell anaemia in Sub-Sahara Africa where an estimated 180,000 children are born each year with this haemoglobinopathy [139].

Since research findings indicate that specified amounts of iron are *sine qua non* for the progression of cell cycle, therefore, iron chelators in addition to ensuring cellular depletion of iron also target critical iron-regulated molecules in the cell cycle to mediate its antiproliferative activity in cancer [140–142].

Phlebotomy may prevent some complications of haemochromatosis and/or diminish their intensity following iron depletion, decrease dyspnoea, pigmentation, fatigue, arthralgia or hepatomegaly, or improved control of diabetes mellitus and left ventricular diastolic function but cannot reverse the course of hepatic cirrhosis, and increased risk of primary liver cancer, hyperthyroidism or hypothyroidism [143]. The challenge of lifetime conventional phlebotomy involving 250–500 ml once or twice weekly during the initial phase, depending on patient characteristics and the level of iron overload, followed by 500 ml every 2–4 months which is considered the best option that is required for normal erythropoiesis and repeated visits to a healthcare facility, and patients' intolerance in some cases [143].

There is a consensus that patients with serial serum ferritin levels exceeding 1000 ng/ml and a total infused red blood cell volume of 120 ml/kg of body weight or more be treated with chelation therapy. Given the myriad of the side effects of classical iron chelators which range from auditory, ocular, and neurological toxicity; growth and skeletal abnormalities, Neutropenia and agranulocytosis; muscle and joint pain; gastric intolerance; hepatic dysfunction; zinc deficiency, gastrointestinal disturbances; rash to possible renal toxicity, natural iron chelators like curcumin, silybin, etc. may be promising alternatives in the management of iron overload in man not only because they are devoid of the side effects of the synthetic iron chelators but because of their multimodal beneficial mechanisms which tend to repair the organs previously damaged by the excess iron.

## 5. Conclusion

Given the rising prevalence of pathologies associated with iron overload in resource poor countries of SSA, natural chelators may be laudable alternatives to both synthetic chelators and phlebotomy in management of iron overload especially in these resource poor countries. There is a need for further studies on the growing iron burden in sickle cell disease together with larger and longer randomized clinical trials need to be performed with natural chelators.

## Figures and Tables

**Table 1:** Selected* in vivo* studies involving natural products/chelators in iron overload.

Natural product	Experimental model/animals	Important phytochemical constituents	Mechanism (s)	Pharmacological effects/findings	References
Curcuminoids	*β*-knockout thalassemic mice with iron overload	*β*-diketone group present in curcuminoids	Iron chelation of plasma nontransferrin bound iron (NTBI)	Decreased levels of NTBI, nonheme iron, and Malondialdehyde (MDA)	[68]

Angel's wings mushroom (*Pleurotus porrigens*)	Iron-overloaded mice	Flavonoid and phenolic acids	Chelation of excess iron	Decrease in plasma Fe^3+^ content	[105]

Green tea extract (GTE)	*β*-knockout thalassemic (BKO) mice diagnosed with iron overload	Epigallocatechin-3-gallate (EGCG)	Anti-oxidation and iron chelation properties	GTE improved liver and pancreatic *β*-cell activity by decreasing redox iron/free radicals	[144]

*Spondias pinnata *bark	Swiss albino mice	Gallic acid (GA) and methyl gallate (MG) isolated from *Spondias pinnata *bark extract	Antioxidation, chelation of free iron, and reducing ferritin-bound iron	Curative effect of GA and MG against iron overload induced hepatic damage	[145]

*Clerodendrum colebrookianum* leaves	Iron-overloaded swiss albino mice	Flavonoid and phenolic acids	Antioxidation and chelating activities	Hepatoprotective effects	[146]

*Nerium indicum *leaves	Iron-overloaded mice	Flavonoid and phenolic compounds	Antioxidant and iron-chelating properties	Decreased iron overload-induced toxicity	[147]

*Terminalia chebula*	Iron-overloaded swiss albino mice	Flavonoid and phenolic compounds	Radical scavenging, chelation, and DNA protective effects	Decreased iron overload-induced toxicity	[148]

*Emblica officinalis* (EO) fruit extract	Iron-overloaded swiss albino mice	Flavonoid and phenolic compounds	Antioxidation and chelation activities	Reduced liver iron, serum ferritin, and serum enzyme levels	[149]

Wild edible fruit of *Prunus nepalensis* Ser. (Steud)	Iron-overloaded swiss albino mice	Purpurin, tannic acid, methyl gallate, reserpine, gallic acid, ascorbic acid, catechin, and rutin	Iron chelating, scavenging, and reducing properties	Amelioration of iron overload-induced hepatotoxicity	[150]

Insectivorous plant (*Drosera burmannii Vahl*.)	Iron-overloaded swiss albino mice	Phenols, flavonoids, carbohydrates, tannins, alkaloids, and ascorbic acid	Iron chelation activity	Reduced liver iron content and reduced liver damage	[151]

*Medicago Sativa *and *Allium Porrum*	Iron-overloaded rats	*Medicago sativa* contains total phenol, flavonoids, alkaloids, coumarins, triterpenes, and phytosterols	Iron chelation activity	Decrease in serum ferritin and iron concentration	[152]
		*Allium porrum* contains carotenoids chlorophyll, glycosides, phenols, and flavonoid			

*Melilotus officinalis*	Iron-overloaded rats	Flavonoids and phenolic compounds	Iron chelation and antioxidant properties	Enhanced excretion of iron in urine and feces with vital organ protective effect	[153]

**Table 2:** Selected natural products with iron chelation/radical scavenging properties (*in vitro *studies).

Natural product	Experimental model	Constituents responsible for activity	Mechanism (s)	Pharmacological effects/findings	References
*Garcinia kola*	Microsomal lipid peroxidation	Kolaviron	Antioxidation and chelating properties	Mitigation of iron/ascorbate-induced damage to microsomal lipids	[97]

*Azadirachta indica *(neem) leaves	Ferric reducing antioxidant plasma (FRAP) assay	Flavonoid and phenolic compounds	Anti-oxidation, scavenging and reduction of the power of ferric ion (Fe^3+^) to ferrous ion (Fe^2+^)	Antioxidant activity	[107]

Lotus (*Nelumbonucifera Gaertn*) leaves	Iron loaded human hepatocellular (HepG2) cells	Polyphenolic compounds	Antioxidation, iron chelating and scavenging properties	Dose-dependent decrease in labile iron pool	[154]

Bergamot and orange juices	Iron overloaded human lung epithelial cells (A549 cells)	Flavonoids	Antioxidation chelating and blockade of the redox activity of iron	Reduced generation of reactive oxygen species and membrane lipid peroxidation. Inhibition of DNA-oxidative damage	[155]

*Spondias pinnata* stem bark	*In vitro* assays (IC_50_ measurements)	Flavonoids and phenolic compounds	Antioxidation, radical scavenging, Iron reducing, and chelating properties	Decrease in the level of labile iron pool	[156]

Essential oils of *Ocimum basilicum *L., *Origanum vulgare *L., and *Thymus vulgaris* L.	*In vitro* assays (IC_50_ measurements)	Essential oils	Antioxidation and chelating properties	Inhibition of lipid peroxidation induced by Fe(2+)/ascorbate or by Fe(2+)/H(2)O(2)	[157]
Red ginger (*Zingiber officinale* var. Rubra) and white ginger (*Zingiber officinale *Roscoe)	Inhibitory effect on Fe^2+^-induced lipid peroxidation in rat brain *in vitro*	Flavonoids and phenolic compounds	Fe(2+) chelating ability, OH scavenging and iron reducing power activities	Protective effect by reducing malondialdehyde contents of the brain	[158]

Unripe pawpaw (*Carica papaya*) fruit	Inhibitory effect on Fe^2+^-induced lipid peroxidation in rat's pancreas *in vitro*	Flavonoids and phenolic compounds	Antioxidation, iron chelating and radical scavenging properties	Reduced malondialdehyde contents in the pancreas; Inhibition of lipid peroxidation	[159]

*Primula heterochroma*	Inhibitory effect on Fe^2+^-induced lipid peroxidation and oxidative stress in rat brain *in vitro*	Flavonoids and phenolic compounds	Fe(2+) chelating, radical scavenging and reducing power activities	Protective effect by reducing brain thiobarbituric acid reactive substances (TBARS) levels	[160]
